# CDC Grand Rounds: Creating a Healthier Future Through Prevention of Child Maltreatment

**Published:** 2014-03-28

**Authors:** Janet Saul, Linda A. Valle, James A. Mercy, Shairi Turner, Rachel Kaufmann, Tanja Popovic

**Affiliations:** 1 Division of Violence Prevention, National Center for Injury Prevention and Control, CDC; 2 Florida Department of Health; 3 Center for Surveillance, Epidemiology, and Laboratory Services, CDC; 4 Office of the Director, CDC

Child maltreatment is abuse or neglect of a child by a parent or other caregiver that results in potential or actual harm or threats of harm to a child (1). Maltreatment encompasses both acts of commission (abuse) and omission (neglect). Child maltreatment is divided into four types: 1) physical abuse (e.g., hitting, kicking, shaking, or burning); 2) sexual abuse (e.g., rape or fondling); 3) psychological abuse (e.g., terrorizing or belittling); and 4) neglect, which involves the failure to meet a child’s basic physical, emotional, or educational needs (e.g., not providing nutrition, shelter, or medical or mental health care) or the failure to supervise the child in a way that ensures safety (e.g., not taking reasonable steps to prevent injury) (1). In 2012, a total of 1,593 children were reported to have died as a result of maltreatment in the United States (2). Also in 2012, state child protective service (CPS) agencies received an estimated 3.4 million reports of alleged maltreatment, involving an estimated 6.3 million children. Following the CPS investigation or other response, nearly 700,000 children were confirmed as having been maltreated (2). However, many cases are never reported to authorities; the actual scope of child maltreatment is greater (3). For example, data from a nationally representative survey in 2011 of children and adult caregivers (usually parents) suggest that 13.8% of children are maltreated each year and 25.6% experienced maltreatment at some point during childhood (4).

Although self-reports suggest that the risk for experiencing any type of maltreatment increases with age (4), children aged <3 years are at greatest risk for severe injuries; approximately 70% of documented child maltreatment deaths occur in this age group (2). Children with special needs, such as chronic illness or disabilities that increase caregiver burden, also appear to be at greater risk for maltreatment (5). Caregiver factors that make maltreatment more likely include stress, inadequate parenting knowledge and skills, and factors that impair judgment, such as substance abuse or depression (6). Family factors include those that produce or exacerbate stress, such as poverty or unemployment, or isolation from support (6). Similarly, predisposing community factors include elevated levels of violence, housing instability, poverty, all factors that undermine safety and stability (7–8).

Child maltreatment results in immediate physical or emotional harm or threat of harm to a child. However, it also affects health across the lifespan by contributing to social, emotional, and cognitive impairments that, in turn, can lead to health risk behaviors and then to disease, injury, disability, and ultimately to early death (Figure). The Adverse Childhood Experiences study of more than 17,000 adult members of the Kaiser Permanente health maintenance organization demonstrated that the number of adverse childhood experiences (defined as physical, sexual, and emotional abuse; emotional and physical neglect; and caregiver risk factors of substance abuse, mental illness, separation or divorce, incarceration, or violent treatment of the mother) is correlated with an increased likelihood of a range of negative outcomes later in life, such as depression, suicide attempts, alcohol and illicit drug abuse, smoking, unintended pregnancies, fetal death, sexually transmitted diseases, obesity, cancer, diabetes, ischemic heart disease (9–10), and premature death (11).

CDC has estimated that the total lifetime economic cost of new child maltreatment cases in the United States in 2008 was approximately $124 billion (12). Of this amount, 69.2% was attributed to lost productivity over the lifetimes of the children, 20.2% was attributed to health-care costs, 3.7% to special education costs, 3.6% to child welfare costs, and 3.2% to criminal justice costs.

## Prevention Challenges and Approaches

There are important gaps in child maltreatment prevention efforts. The first involves the crucial need for ongoing, systematically collected data that are reliable and accurately reflect the true magnitude and nature of this problem. The major existing child maltreatment surveillance system (National Child Abuse and Neglect Data System [2]) only includes cases coming to the attention of CPS agencies; it thus understates the actual incidence of child maltreatment (3), which suggests that the consequences and cost of child maltreatment are underestimated and underappreciated. Surveillance methods are needed that are less dependent on cases coming to the attention of child welfare authorities. Promising approaches for gathering data on child maltreatment include conducting regular, on-going surveys with children and parents and making better use of hospital emergency department discharge codes indicating child maltreatment, child fatality review data, and National Violent Death Reporting System data.

Second, since the 1970s, when child maltreatment became more broadly recognized, societal systems for addressing child maltreatment have been primarily reactive, focusing more on reporting cases, CPS responses, immediate treatment of injuries, and addressing longer-term mental and physical outcomes rather than preventive services. There is a growing evidence base for the effectiveness of strategies to prevent child maltreatment (13). Although addressing the needs of families who have already experienced child maltreatment remains essential, primary prevention of the initial occurrence should receive at least as much emphasis as responses to maltreatment. This would require full engagement of public health and other systems that have the ability to evaluate and implement prevention strategies.

Another important gap is addressing the social context in which child maltreatment occurs. Existing evidence supports strategies that teach and support positive parenting behaviors (13), and efforts are needed to facilitate the widespread adoption and quality implementation of these promising strategies. However, approaches that focus on modifying individual-level and family-level factors (e.g., parenting skills) do not always take into account that both child maltreatment and safe, stable, and nurturing relationships (SSNRs) emerge from and are sustained within social contexts. Various studies have found that social determinants such as neighborhood poverty, housing stress (i.e., instability or vacant housing), and unemployment are associated with child maltreatment (8). Policies and other interventions that have the potential to change the social context in which families function might increase caregivers’ ability to provide SSNRs, and ultimately decrease child maltreatment at the broader population level. Identification, development, and promotion of such interventions will require the efforts of persons in many sectors (e.g., public health, housing, community development, education, and policy).

Commitment to a rigorous science base is critical and demands that development and implementation of programs to promote SSNRs are based on reliable data and sound evidence of effectiveness. When a strong evidence base does not exist, policies could require that publicly funded programs be rigorously evaluated to establish their short-term and long-term outcomes, benefits versus costs, and levels of implementation.

## The Role of Public Health in Preventing Child Maltreatment

As a national public health agency, CDC supports surveillance, research, and programmatic activities aimed at preventing child maltreatment.* For example, CDC worked with child maltreatment professionals, specialists in head trauma caused by abuse, and state health department representatives to develop uniform definitions and methods for using hospital discharge data to monitor head trauma caused by abuse. CDC also developed uniform definitions for child maltreatment and recommended data elements for surveillance to better gauge the scope of the problem, identify groups at high risk, and monitor the effects of prevention programs.

Other CDC efforts are geared toward implementing effective approaches to prevent child maltreatment. For example, in collaboration with not-for-profit partners, CDC is funding two demonstration sites of Triple P (Positive Parenting Program), a system of interventions in which training and support are delivered to meet differing levels of families’ needs. Triple P targets an entire community through media messages, brief consultations with families in primary care and other settings, and more intensive services and counseling to families experiencing problems in parenting or child behavior. Preliminary evidence indicates that Triple P can prevent child maltreatment (14) and is cost beneficial (15). These demonstrations will inform implementation of the Triple P system in communities. CDC also provides consultation to the Health Resources and Services Administration and Administration for Children and Families on the Maternal, Infant, and Early Childhood Home Visitation Program.† Home visitation involves trained practitioners visiting parents in their homes to provide education and support on child development, child care, and parenting skills. The Task Force for Community Preventive Services has stated that home visitation programs can be both effective and cost-beneficial (16). CDC also developed Essentials for Childhood,§ which proposes community strategies for promoting healthy relationships and environments for children.

## The State Public Health Agency’s Role in Prevention

Although a public health role in child maltreatment prevention at the state level is not yet well established, progress is being made. CDC, in partnership with a not-for-profit partner, invested in the Public Health Leadership for Child Maltreatment Prevention Initiative, which worked to raise awareness about child maltreatment as a preventable public health issue and to identify ways to support, improve, and expand child maltreatment prevention efforts in state health departments.¶

One example of state-level work on child maltreatment is in Florida, where the 2007 passage of the Florida Child Abuse Prevention and Permanency Plan legislated the development of a statewide plan on prevention and permanent placement of abused and neglected children, as well as establishment of the multiagency Children and Youth Cabinet that included representation from the leadership of the Department of Health, the Department of Children and Families, the Department of Education, the Department of Juvenile Justice, representatives of child advocacy groups, and other stakeholders. The statewide plan called for integrating support for five protective factors into state systems that serve both parents and children.** These factors are 1) nurturing and attachment, 2) knowledge about parenting and child development, 3) parental resilience, 4) social connections, and 5) concrete support for parents. These principles also are used within the statewide Healthy Start Program, the Teen Parent Program, and other programs for children and parents. In addition, the state Early Periodic Screening, Detection, and Treatment Program includes information on child maltreatment prevention in the literature it provides to families and health-care providers.

Florida’s Department of Health also works with the Department of Children and Families to provide traditional child protection services, including the investigation of suspected cases of abuse and neglect; medical, psychological, and psychosocial evaluations; forensic and specialized interviews; and training for family members and professionals. Finally, the Department of Health houses the Child Abuse Death Review Committee that reviews all cases of children who died as a result of verified maltreatment. The committee works to identify rectifiable deficiencies in the services provided to these children and their families by public and private agencies.

## Conclusion

Child maltreatment is an avoidable tragedy and a preventable public health problem. In addition to the toll on individual children, it has profound negative implications for the entire society. Essential strategies for addressing child maltreatment and ensuring the public’s health include prevention of child maltreatment before it occurs and promotion of children’s healthy development, as well as approaches to ameliorate the effects of child maltreatment. There are evidence-based interventions to prevent child maltreatment, including ones such as home visitation that are within the traditional purview of public health. Working with the public and other agencies, such as those responsible for child welfare, criminal justice, and education, the public health community can be instrumental in developing and disseminating the evidence base for both individual and population-based prevention strategies. Only this coordinated effort can ensure that children never experience child maltreatment but rather have safe, stable, and nurturing relationships during their critical periods of development.

## Figures and Tables

**FIGURE:**
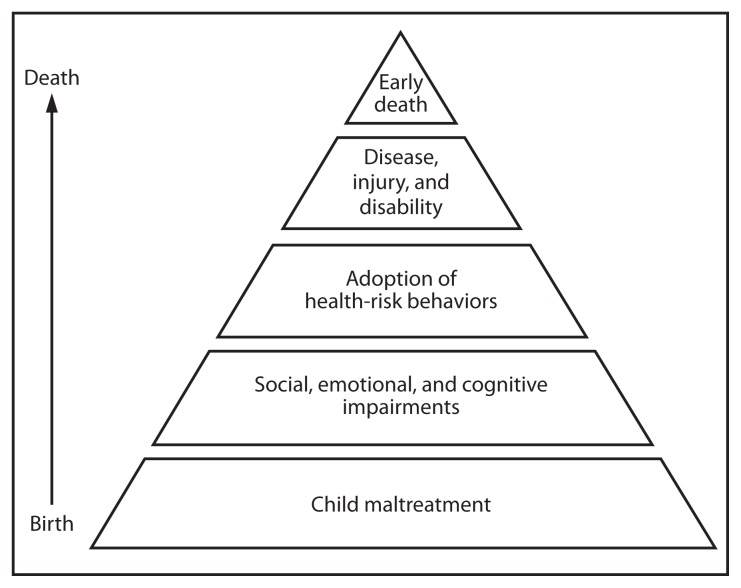
Potential influences of child maltreatment throughout the lifespan **Adapted from:** Felitti VJ, Anda RF, Nordenberg D, et al. Relationship of childhood abuse and household dysfunction to many of the leading causes of death in adults: the Adverse Childhood Experiences (ACE) Study. Am J Prev Med 1998;14:245–58.

## Footnotes

* Additional information available at http://www.cdc.gov/violenceprevention/childmaltreatment/index.html.

† Additional information available at http://mchb.hrsa.gov/programs/homevisiting/index.html.

§ Additional information available at http://www.cdc.gov/violenceprevention/childmaltreatment/essentials/index.html.

¶ Additional information available at http://www.vetoviolence.org/childmaltreatment/phl.

** Additional information available at http://www.flgov.com/wp-content/uploads/childadvocacy/section_1_introduction_june_2012_final.pdf.

## References

[1] Leeb RT, Paulozzi L, Melanson C, Simon T, Arias I (2008). Child maltreatment surveillance: uniform definitions for public health and recommended data elements, version 1.0.

[2] Administration on Children, Youth, and Families, Children’s Bureau (2013). Child maltreatment 2012.

[3] Sedlak AJ, Mettenburg J, Basena M (2010). Fourth national incidence study of child abuse and neglect (NIS-4): report to Congress.

[4] Finkelhor D, Turner HA, Shattuck A, Hamby SL (2013). Violence, crime, and abuse exposure in a national sample of children and youth. JAMA Pediatr.

[5] Algood CL, Hong JS, Gourdine RM, Williams AB (2011). Maltreatment of children with developmental disabilities: an ecological systems analysis. Child Youth Serv Rev.

[6] Stith SM, Liu TL, Davies LC (2009). Risk factors in child maltreatment: a meta-analytic review of the literature. Aggress Violent Behav.

[7] Coulton CJ, Crampton DS, Irwin M, Spilsbury JC, Korbin JE (2007). How neighborhoods influence child maltreatment: a review of the literature and alternative pathways. Child Abuse Negl.

[8] Freisthler B, Merritt DH, LaScala EA (2006). Understanding the ecology of child maltreatment: a review of the literature and directions for future research. Child Maltreat.

[9] Middlebrooks JS, Audage NC (2008). The effects of childhood stress on health across the lifespan.

[10] Felitti VJ, Anda RF, Nordenberg D (1998). Relationship of childhood abuse and household dysfunction to many of the leading causes of death in adults: the Adverse Childhood Experiences (ACE) Study. Am J Prev Med.

[11] Brown DW, Anda RF, Tiemeier H (2009). Adverse childhood experiences and the risk of premature mortality. Am J Prev Med.

[12] Fang X, Brown D, Florence C, Mercy J (2012). The economic burden of child maltreatment in the United States and implications for prevention. Child Abuse Negl.

[13] MacMillan HL, Wathan CN, Barlow J, Fergusson DM, Leventhal JM, Tausig HN (2009). Interventions to prevent child maltreatment and associated impairment. Lancet.

[14] Prinz RJ, Sanders MR, Shapiro CJ, Whitaker DJ, Lutzker JR (2009). Population-based prevention of child maltreatment: the U.S. Triple P system population trial. Prev Sci.

[15] Foster EM, Prinz RJ, Sanders MR, Shapiro CJ (2008). The costs of a public health infrastructure for delivering parenting and family support. Child Youth Serv Rev.

[16] Task Force on Community Preventive Services (2005). Recommendations to reduce violence through early childhood home visitation, therapeutic foster care, and firearms laws. Am J Prev Med.

